# Supplementary material for: A Humanized Yeast Phenomic Model of Deoxycytidine Kinase to Predict Genetic Buffering of Nucleoside Analog Cytotoxicity
Source: Genes (Basel). 2019 Sep 30;10(10):770. doi: 10.3390/genes10100770 (PMC6826991; doi:10.3390/genes10100770)
Supplement: Supplementary file 1 [file genes-10-00770-s001.zip › Additional_Files/Additional_File7_GOTermHeatmaps.zip!/Additional_File7_GOTermHeatmaps/negative_regulation_of_macroautophagy.pdf]

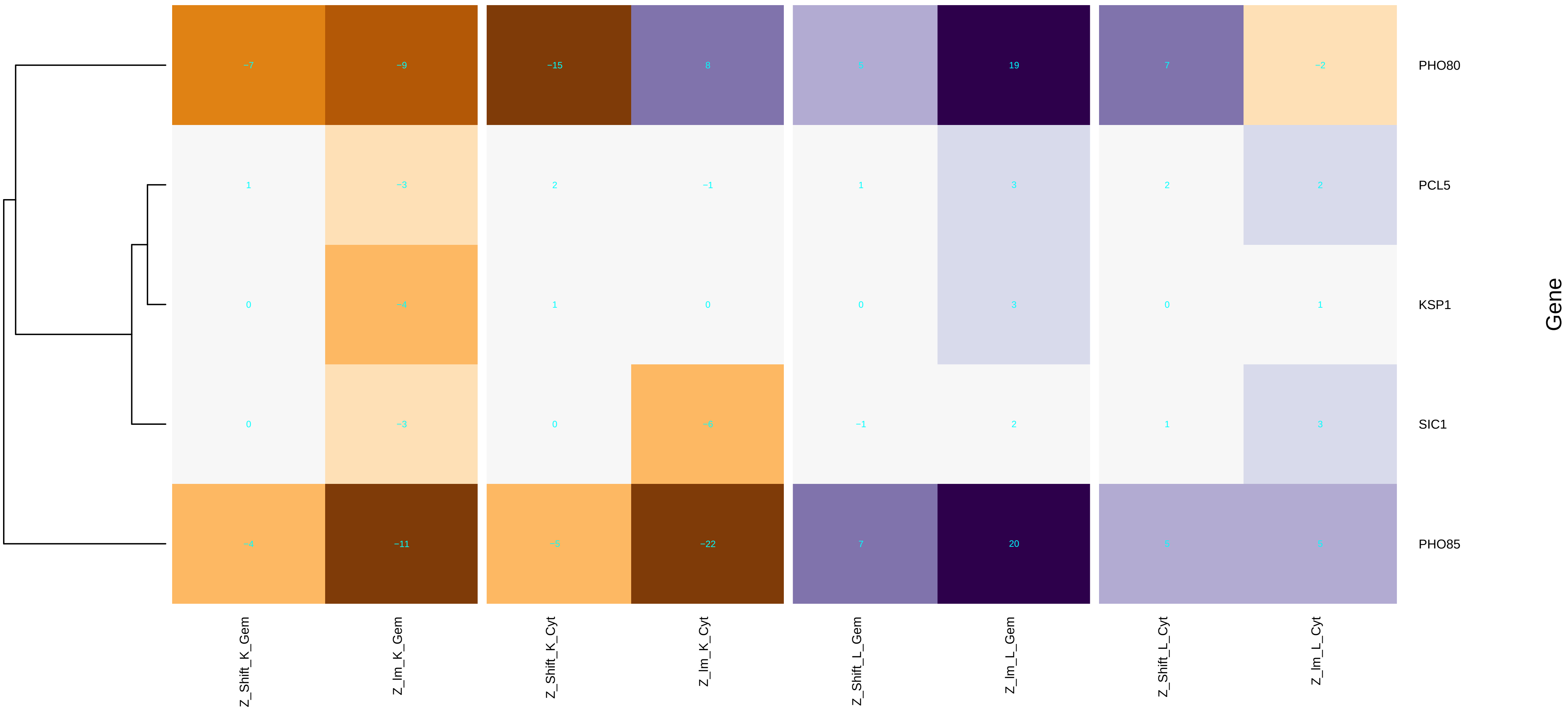
−7 −9 −15 8 5 19 7 −2
PHO80
1 −3 2 −1 1 3 2 2
PCL5
0 −4 1 0 0 3 0 1
KSP1
0 −3 0 −6 −1 2 1 3
SIC1
−4 −11 −5 −22 7 20 5 5
PHO85
Gene
Z_Shift_K_Gem
Z_Im_K_Gem
Z_Shift_K_Cyt
Z_Im_K_Cyt
Z_Shift_L_Gem
Z_Im_L_Gem
Z_Shift_L_Cyt
Z_Im_L_Cyt
